# Supplementary material for: Organized Breast and Cervical Cancer Screening: Attendance and Determinants in Rural China
Source: Int J Environ Res Public Health. 2022 Jul 6;19(14):8237. doi: 10.3390/ijerph19148237 (PMC9318997; doi:10.3390/ijerph19148237)
Supplement: Supplementary file 1 [file ijerph-19-08237-s001.zip › ijerph-1716854-supplementary.pdf]

Supplementary Online Content

Organized Breast and Cervical Cancer Screening: Attendance and Determinants in Rural China

Table S1. Determinants of screening willingness and attendance of breast and cervical cancer screening in rural China 2018-2019-Including adjustment for family support.

Table S2. Determinants of the willingness to participate again and to recommend screening to others of organized breast and cervical cancer screening in rural China 2018-2019-Including adjustment for family support.

Table S3. The association of the resource of notification about screening with the willingness to participate again and to recommend screening to others of organized breast and cervical cancer screening in rural China 2018-2019-Including adjustment for family support.

Table S4. Determinants of screening willingness and attendance of breast and cervical cancer screening in rural China 2018-2019-Adjustment self-reported economic status instead of housing characteristics.

Table S5. Determinants of the willingness to participate again and to recommend screening to others of organized breast and cervical cancer screening in rural China 2018-2019- Adjustment self-reported economic status instead of housing characteristics.

Table S6. The association of the resource of notification about screening with the willingness to participate again and to recommend screening to others of organized breast and cervical cancer screening in rural China 2018-2019- Adjustment self-reported economic status instead of housing characteristics.

This supplementary material has been provided by the authors to give readers additional information about their work.

Table S1. Determinants of screening willingness and attendance of breast and cervical cancer screening in rural China 2018-2019-Including adjustment for family support.

	**Total participants**				**Participants received screening notification**			
	**Screening willingness**		**Attendance**		**Screening willingness**		**Attendance**	
	**Unadjusted OR(95%CI)**	**Adjusted OR(95%CI)**	**Unadjusted OR (95%CI)**	**Adjusted OR(95%CI)**	**Unadjusted OR(95%CI)**	**Adjusted OR(95%CI)**	**Unadjusted OR (95%CI)**	**Adjusted OR(95%CI)**
**Organizer-level factors**								
**Receiving screening notification**								
Yes	2.25(1.83,2.75)***	1.58(1.262,1.979)***	104.21(55.25,196.56)***	97.861(51.337,186.547)***				
No	1.00	1.00	1.00	1.00				
**Receiver lever factors -Sociodemographic characteristics**								
**Age**								
65 years and above	0.296(0.208,0.422)***	0.531(0.347,0.812)	0.275(0.195,0.389)***	0.606(0.368,0.995)	0.363(0.222,0.595)***	0.595(0.332,1.067)	0.395(0.258,0.603)***	0.585(0.353,0.968)*
55-64 years	0.549(0.394,0.766)***	0.78(0.539,1.128)	0.869(0.659,1.147)	1.25(0.846,1.848)	0.540(0.351,0.830)**	0.787(0.486,1.273)	0.840(0.593,1.190)	1.168(0.785,1.738)
45-54 years	0.873(0.620,1.229)	0.913(0.635,1.312)	1.141(0.866,1.502)	1.13(0.786,1.625)	0.829(0.535,1.282)	0.918(0.577,1.461)	0.949(0.673,1.338)	1.058(0.731,1.532)
35-44 years	1.00	1.00	1.00	1.00	1.00	1.00	1.00	1.00
**Education status**								
Senior high schooland above	1.327(0.916,1.921)	0.813(0.539,1.227)	1.173(0.837,1.644)	0.744(0.474,1.168)	1.388(0.845,2.280)	0.939(0.545,1.616)	0.984(0.653,1.483)	0.699(0.443,1.103)
Junior high school	1.880(1.491,2.370)***	1.379(1.061,1.793)**	1.545(1.266,1.885)***	1.18(0.894,1.56)	1.747(1.293,2.361)***	1.43(1.02,2.005)*	1.454(1.132,1.866)**	1.226(0.923,1.627)
Primary and lower	1.00	1.00	1.00	1.00	1.00	1.00	1.00	1.00
**Marital status**								
Married	1.995(1.503,2.647)***	1.163(0.834,1.621)	2.642(1.908,3.658)***	1.303(0.839,2.025)	2.077(1.375,3.138)***	1.339(0.834,2.149)	2.015(1.359,2.986)***	1.34(0.857,2.094)
Single/Divorced/Widowed/Other	1.00	1.00	1.00	1.00	1.00	1.00	1.00	1.00
**Housing characteristic**								
Three-story house or larger	0.978(0.628,1.525)	0.583(0.378,0.901)*	0.764(0.538,1.085)	0.74(0.46,1.188)	0.731(0.433,1.235)	0.55(0.313,0.968)*	0.848(0.546,1.319)	0.687(0.425,1.111)
Two-story	0.827(0.557,1.228)	0.591(0.399,0.877)**	0.808(0.588,1.110)	0.7(0.458,1.071)	0.782(0.485,1.260)	0.639(0.384,1.063)	0.814(0.547,1.210)	0.693(0.451,1.066)
One-story	0.774(0.539,1.111)	0.817(0.508,1.312)	1.003(0.681,1.478)	0.918(0.553,1.525)	0.898(0.504,1.599)	0.814(0.444,1.493)	0.964(0.596,1.559)	0.925(0.553,1.546)
Tiled-roof house	1.00	1.00	1.00	1.00	1.00	1.00	1.00	1.00
**Family Support**								
Somewhat high/Very high	1.369(1.108,1.692)**	1.132(0.793,1.616)	1.562(1.283,1.902)***	1.088(0.748,1.581)	1.664(1.097,2.525)*	1.136(0.716,1.802)	1.476(1.047,2.080)*	1.11(0.757,1.626)
Neutral	1.792(1.299,2.472)***	1.073(0.847,1.359)	1.821(1.383,2.398)***	1.036(0.794,1.351)	1.250(0.945,1.655)	1.016(0.744,1.386)	1.224(0.959,1.562)	1.012(0.773,1.324)
Very low/Somewhat low	1.00	1.00	1.00	1.00	1.00	1.00	1.00	1.00
**Time/day online with a mobile phone**								
60 mins and above	1.687(1.355,2.099)***	1.206(0.936,1.554)	1.249(1.032,1.510)*	0.993(0.754,1.307)	1.511(1.134,2.014)**	1.054(0.757,1.468)	1.209(0.952,1.534)	0.961(0.727,1.271)
0-59 mins	1.00	1.00	1.00	1.00	1.00	1.00	1.00	1.00
**Receiver lever factors-Disease-related characteristics**								
**Seeing doctor for gynecological problems**								
Yes	2.693(2.105,3.446)***	1.738(1.317,2.295)***	2.439(2.002,2.972)***	2.153(1.619,2.862)***	3.018(2.163,4.209)***	2.031(1.413,2.921)***	2.675(2.062,3.471)***	2.049(1.536,2.735)***
No	1.00	1.00	1.00	1.00	1.00	1.00	1.00	1.00
**Gynecological physical examination on one's own initiative**								
Yes	2.620(2.088,3.288)***	1.595(1.228,2.071)***	3.035(2.506,3.676)***	2.135(1.638,2.784)***	3.00(2.226,4.045)***	2.074(1.494,2.88)***	2.716(2.134,3.458)***	2.091(1.599,2.736)***
No	1.00	1.00	1.00	1.00	1.00	1.00	1.00	1.00
**Experiencing symptoms/discomfort (irregular vaginal bleeding, nipple discharge, breast lump, abnormal leukorrhea or similar)**								
Yes	1.554(1.276,1.893)***	1.458(1.178,1.804)***	0.939(0.783,1.126)	0.755(0.594,0.961)*	1.418(1.091,1.842)**	1.331(1.007,1.76)*	0.880(0.701,1.103)	0.802(0.629,1.024)
No	1.00	1.00	1.00	1.00	1.00	1.00	1.00	1.00
**Heard of two-cancer**								
Yes	2.344(1.901,2.889)***	1.583(1.251,2.003)***	2.661(2.136,3.313)***	1.314(0.975,1.771)	2.166(1.605,2.923)***	1.683(1.214,2.333)**	1.470(1.114,1.939)**	1.256(0.926,1.703)
No	1.00	1.00	1.00	1.00	1.00	1.00	1.00	1.00
**Relatives or friends suffered from two-cancer in the past 5 years**								
Yes	1.355(1.010,1.817)*	1.001(0.728,1.377)	1.071(0.829,1.382)	0.834(0.597,1.166)	1.237(0.845,1.812)	0.924(0.613,1.392)	0.963(0.703,1.318)	0.817(0.582,1.147)
No	1.00	1.00	1.00	1.00	1.00	1.00	1.00	1.00

Table S2. Determinants of the willingness to participate again and to recommend screening to others of organized breast and cervical cancer screening in rural China 2018-2019-Including adjustment for family support.

	**Willingness to participate again**		**Willingness to recommend screening to others**	
	**Unadjusted OR(95%CI)**	**Adjusted OR(95%CI)**	**Unadjusted OR (95%CI)**	**Adjusted OR(95%CI)**
**Organizer lever factors**				
**Travel time to the hospital**				
≥30 min	0.977(0.554,1.722)	0.833(0.41,1.691)	1.071(0.668,1.718)	1.113(0.622,1.991)
15–29 min	1.746(0.756,4.033)	1.475(0.563,3.863)	1.829(0.927,3.608)	1.649(0.767,3.547)
<15 min	1.00	1.00	1.00	1.00
**Received report within 2 weeks of screening**				
Yes	0.939(0.552,1.597)	0.79(0.393,1.587)	0.967(0.626,1.495)	0.898(0.521,1.547)
No	1.00	1.00	1.00	1.00
**Received report as of now**				
Yes	1.075(0.544,2.123)	0.933(0.383,2.273)	0.821(0.450,1.499)	0.575(0.269,1.228)
No	1.00	1.00	1.00	1.00
**Continuity of screening**				
Yes	1.210(0.701,2.090)	0.832(0.416,1.663)	1.767(1.099,2.843)*	1.412(0.799,2.497)
No	1.00	1.00	1.00	1.00
**Overall perceived smoothness**				
Yes	2.570(1.501,4.401)***	1.571(0.801,3.08)	1.690(1.057,2.702)*	1.38(0.78,2.44)
No	1.00	1.00	1.00	1.00
**Overall perceived usefulness**				
Yes	7.513(4.374,12.905)***	7.936(4.138,15.217)***	3.513(2213,5.578)***	3.751(2.132,6.599)***
No	1.00	1.00	1.00	1.00
**Providers lever factors**				
**Waiting time in the hospital**				
≥30 min	1.044(0.556,1.962)	1.106(0.519,2.36)	1.317(0.798,2.173)	1.266(0.702,2.285)
15–29 min	0.940(0.345,2.560)	1.001(0.317,3.156)	1.014(0.457,2.250)	0.817(0.336,1.987)
<15 min	1.00	1.00	1.00	1.00
**Doctor's explanations unclear**				
Very low/Somewhat low	0.848(0.445,1.618)	1.008(0.473,2.149)	0.737(0.430,1.262)	0.706(0.377,1.321)
Neutral	0.376(0.146,0.967)*	0.721(0.225,2.309)	0.432(0.186,1.004)	0.641(0.237,1.733)
Somewhat high/Very high	1.00	1.00	1.00	1.00
**Ward cleanliness**				
Somewhat high/Very high	3.750(0.867,16.220)	5.264(0.863,32.1)	1.238(0.251,6.117)	0.908(0.147,5.596)
Neutral	2.969(0.803,10.978)	3.385(0.62,18.481)	1089(0.239,4.956)	0.888(0.155,5.106)
Very low/Somewhat low	1.00	1.00	1.00	1.00
**Ward quietness**				
Somewhat high/Very high	2.800(0.919,8.527)	2.279(0.61,8.51)	2.187(0.810,5.908)	2.585(0.827,8.084)
Neutral	1.408(0.683,2.903)	1.248(0.488,3.189)	0.883(0.449,1.736)	0.786(0.349,1.767)
Very low/Somewhat low	1.00	1.00	1.00	1.00
**Presence of others during examination**				
No	1.046(0.621,1.761)	1.119(0.615,2.036)	0.999(0.652,1.533)	1.199(0.736,1.954)
Yes	1.00	1.00	1.00	1.00
**Received health education**				
Yes	1.342(0.799,2.254)	0.933(0.479,1.818)	1.982(1.292,3.041)**	1.441(0.854,2.433)
No	1.00	1.00	1.00	1.00
**Receiver lever factors -Sociodemographic characteristics**				
**Age**				
65 years and above	0.624(0.207,1.88)	1.058(0.247,4.532)	0.395(0.152,1.028)	0.76(0.237,2.435)
55-64 years	0.588(0.258,1.343)	0.584(0.207,1.648)	0.321(0.152,0.677)**	0.384(0.161,0.916)*
45-54 years	0.745(0.325,1.704)	0.895(0.342,2.344)	0.615(0.285,1.328)	0.695(0.3,1.611)
35-44 years	1.00	1.00	1.00	1.00
**Education status**				
Senior high school and above	2.223(0.668,7.399)	2.484(0.612,10.09)	1.941(0.748,5.039)	1.26(0.401,3.96)
Junior high school	1.858(1.026,3.364)*	1.594(0.773,3.287)	1.412(0.887,2.246)	0.893(0.509,1.569)
Primary and lower	1.00	1.00	1.00	1.00
**Marital status**				
Married	1.230(0.470,3.219)	0.952(0.296,3.056)	1.987(0.981,4.025)	1.229(0.522,2.893)
Single/Divorced/Widowed/Other	1.00	1.00	1.00	1.00
**Housing characteristic**				
Three-story house or larger	0.458(0.149,1.41)	0.357(0.099,1.29)	1.212(0.563,2.611)	1.322(0.538,3.246)
Two-story	0.554(0.191,1.604)	0.507(0.151,1.705)	1.291(0.652,2.555)	1.369(0.612,3.061)
One-story	0.901(0.245,3.307)	0.858(0.204,3.604)	1.103(0.485,2.511)	0.978(0.386,2.48)
Tiled-roof house	1.00	1.00	1.00	1.00
**Time/day online with a mobile phone**				
60 mins and above	1.173(0.679,2.026)	1.025(0.512,2.05)	1.525(0.957,2.430)	1.051(0.593,1.864)
0-59 mins	1.00	1.00	1.00	1.00
**Family Supports**				
Somewhat high/Very high	1.272(0.601,2.695)	0.956(0.38,2.405)	3.060(1.464,6.395)**	2.541(1.087,5.939)*
Neutral	1.348(0.766,2.372)	1.429(0.722,2.83)	2.041(1.285,3.241)**	1.864(1.092,3.182)*
Very low/Somewhat low	1.00	1.00	1.00	1.00
**Receiver lever factors-Disease-related characteristics**				
**Seeing a doctor for gynecological problems**				

Yes	1.543(0.888,2.678)	1.881(0.949,3.728)	1.225(0.790,1.901)	0.963(0.559,1.657)
No	1.00	1.00	1.00	1.00
**Gynecological physical examination on one's own initiative**				
Yes	1.246(0.743,2.091)	0.959(0.511,1.8)	1.394(0.909,2.137)	1.126(0.669,1.896)
No	1.00	1.00	1.00	1.00
**Experiencing symptoms/discomfort (irregular vaginal bleeding, nipple discharge, breast lump, abnormal leukorrhea or similar)**				
Yes	0.918(0.546,1,542)	0.884(0.481,1.626)	1.237(0.809,1.893)	1.132(0.694,1.846)
No	1.00	1.00	1.00	1.00
**Heard of two-cancer**				
Yes	2.567(1.464,4.50)***	1.665(0.845,3.279)	3.513(2.213,5.578)***	2.219(1.29,3.816)**
No	1.00	1.00	1.00	1.00
**Relatives or friends suffered from two-cancer in the past 5 years**				
Yes	2.721(0.968,7.648)	2.13(0.689,6.582)	2.943(1.256,6.899)*	2.26(0.906,5.638)
No	1.00	1.00	1.00	1.00
**History of previous screening**				
Yes	2.259(1.340,3.809)**	1.92(1.005,3.666)*	1.863(1.215,2.855)**	1.524(0.91,2.553)
No	1.00	1.00	1.00	1.00

Table S3. The association of the resource of notification about screening with the willingness to participate again and to recommend screening to others of organized breast and cervical cancer screening in rural China 2018-2019-Including adjustment for family support

Resource of notification about screening	n(%)	Willingness to participate again		Willingness recommend screening to others	
		Unadjusted OR (95%CI)	Adjusted OR(95%CI)	Unadjusted OR (95%CI)	Adjusted OR(95%CI)
Other community leaders	300(40.00)	2.37(1.09,5.13)*	3.10(1.19,8.08)*	2.82(1.44,5.55)**	4.25(1.85,9.76)***
Community women's leaders	347(46.27)	2.64(1.23,5.70)*	2.78(1.09,7.05)*	1.95(1.03,3.70)*	2.25(1.04,4.90)*
Friends or relatives	36(4.80)	1.22(0.39,3.83)	1.47(0.35,6.12)	2.51(0.77,8.18)	3.93(0.99,15.54)*
GPs	67(8.93)	1.00	1.00	1.00	1.00

Note: control variables: Control variables: travel time to the hospital, received report within 2 weeks of screening, received report as of now, continuity of screening, overall perceived smoothness, overall perceived usefulness, waiting time in the hospital, doctor's explanations unclear, ward cleanliness, ward quietness, presence of others during examination, received health education, age, education status, marital status, housing characteristic, time/day online with a mobile phone, family supports, seeing a doctor for gynecological problems, gynecological physical examination on one's own initiative, experiencing symptoms/discomfort, heard of two-cancer, relatives or friends suffered from two-cancer in the past 5 years, history of previous screening; *p<0.05, ** p<0.01, *** p<0.001

Table S4. Determinants of screening willingness and attendance of breast and cervical cancer screening in rural China 2018-2019-Adjustment self-reported economic status instead of housing characteristics.

	**Total participants**				**Participants received screening notification**			
	**Screening willingness**		**Attendance**		**Screening willingness**		**Attendance**	
	**Unadjusted OR(95%CI)**	**Adjusted OR(95%CI)**	**Unadjusted OR (95%CI)**	**Adjusted OR(95%CI)**	**Unadjusted OR(95%CI)**	**Adjusted OR(95%CI)**	**Unadjusted OR (95%CI)**	**Adjusted OR(95%CI)**
**Organizer-level factors**								
**Receiving screening notification**								
Yes	2.246(1.834,2.749)***	1.646(1.315,2.059)***	104.21(55.253,196.56)***	99.489(52.229,189.512)***				
No	1.00	1.00	1.00	1.00				
**Receiver lever factors -Sociodemographic characteristics**								
**Age**								
65 years and above	0.296(0.208,0.422)***	0.569(0.373,0.87)**	0.275(0.195,0.389)***	0.637(0.389,1.045)	0.363(0.222,0.595)***	0.675(0.376,1.211)	0.395(0.258,0.603)***	0.626(0.379,1.034)
55-64 years	0.549(0.394,0.766)***	0.786(0.544,1.137)	0.869(0.659,1.147)	1.258(0.852,1.856)	0.540(0.351,0.830)**	0.822(0.508,1.33)	0.840(0.593,1.190)	1.183(0.796,1.759)
45-54 years	0.873(0.620,1.229)	0.914(0.635,1.316)	1.141(0.866,1.502)	1.137(0.791,1.636)	0.829(0.535,1.282)	0.959(0.602,1.527)	0.949(0.673,1.338)	1.073(0.741,1.553)
35-44 years	1.00	1.00	1.00	1.00	1.00	1.00	1.00	1.00
**Education status**								
Senior high school and above	1.327(0.916,1.921)	0.845(0.559,1.278)	1.173(0.837,1.644)	0.748(0.476,1.174)	1.388(0.845,2.280)	0.976(0.566,1.68)	0.984(0.653,1.483)	0.709(0.45,1.118)
Junior high school	1.880(1.491,2.370)***	1.426(1.097,1.855)**	1.545(1.266,1.885)***	1.162(0.884,1.529)	1.747(1.293,2.361)***	1.45(1.038,2.025)*	1.454(1.132,1.866)**	1.205(0.912,1.592)
Primary and lower	1.00	1.00	1.00	1.00	1.00	1.00	1.00	1.00
**Marital status**								
Married	1.995(1.503,2.647)***	1.222(0.882,1.692)	2.642(1.908,3.658)***	1.351(0.879,2.079)	2.077(1.375,3.138)***	1.455(0.916,2.309)	2.015(1.359,2.986)***	1.383(0.894,2.139)
Single/Divorced/Widowed/Other	1.00	1.00	1.00	1.00	1.00	1.00	1.00	1.00
**Self-reported economic status**								
Somewhat Good/Good	0.641(0.418,0.983)*	0.768(0.484,1.219)	0.851(0.574,1.263)	0.826(0.493,1.384)	0.617(0.348,1.093)	0.641(0.348,1.18)	0.834(0.514,1.354)	0.782(0.464,1.316)
Neutral	0.628(0.469,0.840)**	0.583(0.426,0.799)***	0.984(0.764,1.268)	0.9(0.644,1.257)	0.630(0.427,0.929)*	0.584(0.385,0.886)*	0.982(0.718,1.344)	0.883(0.629,1.24)
Somewhat Bad	0.836(0.603,1.158)	0.847(0.598,1.199)	1.007(0.759,1.335)	0.973(0.672,1.408)	0.831(0.536,1.289)	0.788(0.496,1.252)	1.116(0.784,1.589)	1.001(0.687,1.457)
Bad	1.00	1.00	1.00	1.00	1.00	1.00	1.00	1.00
**Time/day online with a mobile phone**								
60 mins and above	1.687(1.355,2.099)***	1.226(0.951,1.579)	1.249(1.032,1.510)*	1.003(0.764,1.318)	1.511(1.134,2.014)**	1.081(0.777,1.503)	1.209(0.952,1.534)	0.973(0.738,1.284)
0-59 mins	1.00	1.00	1.00	1.00	1.00	1.00	1.00	1.00
**Receiver lever factors-Disease-related characteristics**								
**Seeing doctor for gynecological problems**								
Yes	2.693(2.105,3.446)***	1.763(1.336,2.328)***	2.439(2.002,2.972)***	2.155(1.622,2.863)***	3.018(2.163,4.209)***	2.034(1.416,2.921)***	2.675(2.062,3.471)***	2.046(1.535,2.729)***
No	1.00	1.00	1.00	1.00	1.00	1.00	1.00	1.00
**Gynecological physical examination on one's own initiative**								
Yes	2.620(2.088,3.288)***	1.609(1.24,2.087)***	3.035(2.506,3.676)***	2.137(1.642,2.783)***	3.00(2.226,4.045)***	2.096(1.511,2.906)***	2.716(2.134,3.458)***	2.098(1.606,2.741)***
No	1.00	1.00	1.00	1.00	1.00	1.00	1.00	1.00
**Experiencing symptoms/discomfort (irregular vaginal bleeding, nipple discharge, breast lump, abnormal leukorrhea or similar)**								
Yes	1.554(1.276,1.893)***	1.403(1.133,1.739)**	0.939(0.783,1.126)	0.745(0.584,0.95)**	1.418(1.091,1.842)**	1.257(0.948,1.667)	0.880(0.701,1.103)	0.788(0.616,1.009)
No	1.00	1.00	1.00	1.00	1.00	1.00	1.00	1.00
**Heard of two-cancer**								
Yes	2.344(1.901,2.889)***	1.566(1.237,1.981)***	2.661(2.136,3.313)***	1.316(0.977,1.772)	2.166(1.605,2.923)***	1.68(1.212,2.328)**	1.470(1.114,1.939)**	1.258(0.928,1.705)
No	1.00	1.00	1.00	1.00	1.00	1.00	1.00	1.00
**Relatives or friends suffered from two-cancer in the past 5 years**								
Yes	1.355(1.010,1.817)*	0.973(0.707,1.338)	1.071(0.829,1.382)	0.815(0.584,1.138)	1.237(0.845,1.812)	0.899(0.596,1.354)	0.963(0.703,1.318)	0.797(0.568,1.118)
No	1.00	1.00	1.00	1.00	1.00	1.00	1.00	1.00

Table S5. Determinants of the willingness to participate again and to recommend screening to others of organized breast and cervical cancer screening in rural China 2018-2019- Adjustment self-reported economic status instead of housing characteristics.

	Willingness to participate again		Willingness to recommend screening to others	
	Unadjusted OR(95%CI)	Adjusted OR(95%CI)	Unadjusted OR (95%CI)	Adjusted OR(95%CI)
**Organizer lever factors**				
**Travel time to the hospital**				
≥30 min	0.977(0.554,1.722)	0.895(0.443,1.806)	1.071(0.668,1.718)	0.985(0.555,1.749)
15–29 min	1.746(0.756,4.033)	1.592(0.612,4.143)	1.829(0.927,3.608)	1.622(0.763,3.448)
<15 min	1.00	1.00	1.00	1.00
**Received report within 2 weeks of screening**				
Yes	0.939(0.552,1.597)	0.788(0.396,1.566)	0.967(0.626,1.495)	0.923(0.538,1.585)
No	1.00	1.00	1.00	1.00
**Received report as of now**				
Yes	1.075(0.544,2.123)	0.885(0.364,2.153)	0.821(0.450,1.499)	0.6(0.282,1.276)
No	1.00	1.00	1.00	1.00
**Continuity of screening**				
Yes	1.210(0.701,2.090)	0.915(0.463,1.805)	1.767(1.099,2.843)*	1.373(0.784,2.407)
No	1.00	1.00	1.00	1.00
**Overall perceived smoothness**				
Yes	2.570(1.501,4.401)***	1.573(0.802,3.083)	1.690(1.057,2.702)*	1.378(0.78,2.436)
No	1.00	1.00	1.00	1.00
**Overall perceived usefulness**				
Yes	7.513(4.374,12.905)***	8.166(4.292,15.537)***	3.513(2213,5.578)***	3.628(2.088,6.305)***
No	1.00	1.00	1.00	1.00
**Providers lever factors**				
**Waiting time in the hospital**				
≥30 min	1.044(0.556,1.962)	1.12(0.526,2.386)	1.317(0.798,2.173)	1.221(0.681,2.191)
15–29 min	0.940(0.345,2.560)	0.969(0.309,3.043)	1.014(0.457,2.250)	0.87(0.359,2.109)
<15 min	1.00	1.00	1.00	1.00
**Doctor's explanations unclear**				
Very low/Somewhat low	0.848(0.445,1.618)	0.995(0.473,2.093)	0.737(0.430,1.262)	0.645(0.349,1.193)
Neutral	0.376(0.146,0.967)*	0.776(0.245,2.462)	0.432(0.186,1.004)	0.624(0.233,1.667)
Somewhat high/Very high	1.00	1.00	1.00	1.00
**Ward cleanliness**				
Somewhat high/Very high	3.750(0.867,16.220)	5.01(0.826,30.381)	1.238(0.251,6.117)	0.966(0.154,6.07)
Neutral	2.969(0.803,10.978)	3.44(0.63,18.795)	1089(0.239,4.956)	0.979(0.166,5.782)
Very low/Somewhat low	1.00	1.00	1.00	1.00
**Ward quietness**				
Somewhat high/Very high	2.800(0.919,8.527)	2.653(0.716,9.831)	2.187(0.810,5.908)	2.281(0.735,7.081)
Neutral	1.408(0.683,2.903)	1.202(0.472,3.061)	0.883(0.449,1.736)	0.822(0.363,1.864)
Very low/Somewhat low	1.00	1.00	1.00	1.00
**Presence of others during examination**				
No	1.046(0.621,1.761)	1.16(0.642,2.097)	0.999(0.652,1.533)	1.153(0.715,1.859)
Yes	1.00	1.00	1.00	1.00
**Received health education**				
Yes	1.342(0.799,2.254)	0.99(0.51,1.923)	1.982(1.292,3.041)**	1.422(0.843,2.399)
No	1.00	1.00	1.00	1.00
**Receiver lever factors -Sociodemographic characteristics**				
**Age**				
65 years and above	0.624(0.207,1.88)	1.139(0.274,4.73)	0.395(0.152,1.028)	0.62(0.193,1.991)
55-64 years	0.588(0.258,1.343)	0.688(0.249,1.903)	0.321(0.152,0.677)**	0.374(0.157,0.895)*
45-54 years	0.745(0.325,1.704)	0.992(0.383,2.572)	0.615(0.285,1.328)	0.708(0.305,1.643)
35-44 years	1.00	1.00	1.00	1.00
**Education status**				
Senior high school and above	2.223(0.668,7.399)	2.433(0.602,9.832)	1.941(0.748,5.039)	1.292(0.416,4.006)
Junior high school	1.858(1.026,3.364)*	1.46(0.712,2.992)	1.412(0.887,2.246)	1.018(0.583,1.775)
Primary and lower	1.00	1.00	1.00	1.00
**Marital status**				
Married	1.230(0.470,3.219)	1.102(0.352,3.45)	1.987(0.981,4.025)	1.738(0.746,4.051)
Single/Divorced/Widowed/Other	1.00	1.00	1.00	1.00
**Self-reported economic status**				
Somewhat Good/Good	0.997(0.333,2.980)	1.029(0.291,3.644)	1.630(0.515,5.159)	1.827(0.52,6.426)
Neutral	1.054(0.527,2.108)	0.907(0.399,2.062)	0.743(0.404,1.365)	0.682(0.34,1.369)
Somewhat Bad	1.392(0.614,3.156)	1.234(0.481,3.163)	0.953(0.477,1.904)	0.905(0.416,1.968)
Bad	1.00	1.00	1.00	1.00
**Time/day online with a mobile phone**				
60 mins and above	1.173(0.679,2.026)	0.983(0.498,1.941)	1.525(0.957,2.430)	1.165(0.664,2.045)
0-59 mins	1.00	1.00	1.00	1.00
**Receiver lever factors- Disease-related characteristics**				
**Seeing a doctor for gynecological problems**				
Yes	1.543(0.888,2.678)	1.924(0.974,3.803)	1.225(0.790,1.901)	0.952(0.556,1.633)
No	1.00	1.00	1.00	1.00
**Gynecological physical examination on one's own initiative**				
Yes	1.246(0.743,2.091)	0.924(0.49,1.742)	1.394(0.909,2.137)	1.144(0.682,1.919)
No	1.00	1.00	1.00	1.00

**Experiencing symptoms/discomfort (irregular vaginal bleeding, nipple discharge, breast lump, abnormal leukorrhea or similar)**				
Yes	0.918(0.546,1,542)	0.892(0.483,1.648)	1.237(0.809,1.893)	1.07(0.654,1.751)
No	1.00	1.00	1.00	1.00
**Heard of two-cancer**				
Yes	2.567(1.464,4.50)***	1.638(0.831,3.23)	3.513(2.213,5.578)***	2.371(1.385,4.059)**
No	1.00	1.00	1.00	1.00
**Relatives or friends suffered from two-cancer in the past 5 years**				
Yes	2.721(0.968,7.648)	2.115(0.688,6.5)	2.943(1.256,6.899)*	2.319(0.935,5.75)
No	1.00	1.00	1.00	1.00
**History of previous screening**				
Yes	2.259(1.340,3.809)**	1.857(0.977,3.527)	1.863(1.215,2.855)**	1.535(0.918,2.568)
No	1.00	1.00	1.00	1.00

Table S6. The association of the resource of notification about screening with the willingness to participate again and to recommend screening to others of organized breast and cervical cancer screening in rural China 2018-2019-Adjustment self-reported economic status instead of housing characteristics.

**Resource of notification about screening**	**n(%)**	**Willingness to participate again**		**Willingness recommend screening to others**	
		**Unadjusted OR (95%CI)**	**Adjusted OR(95%CI)**	**Unadjusted OR (95%CI)**	**Adjusted OR(95%CI)**
Other community leaders	300(40.00)	2.37(1.09,5.13)*	3.19(1.23,8.29)*	2.82(1.44,5.55)**	3.66(1.64,8.17)*
Community women's leaders	347(46.27)	2.64(1.23,5.70)*	3.04(1.19,7.76)*	1.95(1.03,3.70)*	2.00(0.94,4.24)
Friends or relatives	36(4.80)	1.22(0.39,3.83)	1.47(0.37,5.91)	2.51(0.77,8.18)	2.93(0.75,11.43)
GPs	67(8.93)	1.00	1.00	1.00	1.00

Note: control variables: travel time to the hospital, received report within 2 weeks of screening, received report as of now, continuity of screening, overall perceived smoothness, overall perceived usefulness, waiting time in the hospital, doctor's explanations unclear, ward cleanliness, ward quietness, presence of others during examination, received health education, age, education status, marital status, self-reported economic status, time/day online with a mobile phone, seeing a doctor for gynecological problems, gynecological physical examination on one's own initiative, experiencing symptoms/discomfort, heard of two-cancer, relatives or friends suffered from two-cancer in the past 5 years, history of previous screening； *p<0.05, ** p<0.01, *** p<0.001
